# Multiple Brillouin zone folding based broadband topological slow light in valley photonic crystals

**DOI:** 10.1016/j.fmre.2025.09.019

**Published:** 2025-10-01

**Authors:** Min Zhang, Tianji Liu, Wei Li

**Affiliations:** a GPL Photonics Laboratory, State Key Laboratory of Luminescence Science and Technology, Changchun Institute of Optics, Fine Mechanics and Physics, Chinese Academy of Sciences, Changchun 130033, China; b University of Chinese Academy of Sciences, Beijing 100039, China

**Keywords:** Topological valley photonic crystals, Broadband topological slow light, Brillouin zone folding, Complementary waveguide, Frequency-selective beam splitter

## Abstract

Topological photonic crystals (TPCs) have attracted considerable attention in integrated photonics community for the intriguing prospect of robust light transport. As a photonic analogue of quantum valley Hall insulators, valley photonic crystals (VPCs) support topologically protected directional edge modes without broken time-reversal symmetry. However, the observation of VPCs-based broadband slow light remains challenging due to the frequency discontinuity of valley kink modes resulting from the broken symmetry at the domain wall. In this work, we achieve continuous broadband topological slow light in two-dimensional VPCs. Based on the coupling of size-perturbed resonator periodic array, the resulting kink mode has been dramatically slow down through multiple Brillouin zone folding and separated with mini-gaps. To circumvent the mini-gaps issue, we propose a continuous broadband topological slow light waveguide consisting of two combined and frequency complementary VPCs waveguides. We also obtain a novel topological frequency-selective beam splitter based on the valley-contrasting directional slow-light propagation. Our results provide exciting opportunities in topological slow-light applications.

## Graphical abstract

**Figure:**
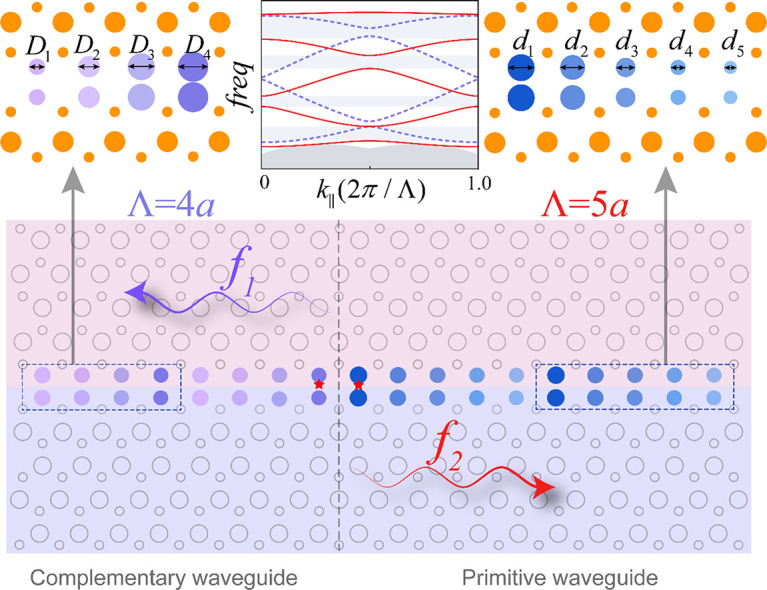

## 1 Introduction

The advent and development of topological photonics [[1], [2], [3], [4], [5]] have offered a valuable approach to addressing the susceptibility of optical devices to disorder and defects, innovating the mainstream photonic structures such as waveguide arrays [6], photonic crystals [[7], [8]], cavities [9], and metamaterials [[10], [11], [12], [13], [14], [15]]. In particular, topological photonic crystals (TPCs) [[16], [17], [18], [19]], which is the photonic analogue of topological insulators in condensed matter systems [[20], [21], [22], [23], [24], [25], [26], [27]], disclose a plethora of novel topological phases with different dimensions [[28], [29], [30], [31], [32]] and higher orders [[33], [34], [35], [36], [37], [38]]. Amongst TPCs, valley photonic crystals (VPCs), exploiting the valley degree of freedom in time-reversal invariant systems, offer a valuable topological protection for a range of on-chip photonic structures [[39], [40]]. Specifically, as one of the promising and practical application fields in topological photonics [41], TPCs including VPC-based slow light waveguides attract much attention in recent years [[42], [43], [44]].

Slow-light devices can enhance the light-matter interaction and significantly scale down the photonic device footprint [45]. However, conventional slow-light devices have suffered from two fundamental limitations [46]: (1) slow-light devices are sensitive to disorders from fabrication imperfections which cause the propagation-loss from undesired backscattering and even Anderson localization [47]; (2) the reduction of group velocity is typically accompanied by decreasing in bandwidth, resulting in narrow bandwidths and limited on-chip performance [43]. To address two issues, based on the Brillouin zone folding strategy, broadband topological slow light has been recently theoretically and experimentally demonstrated in photonic structures with broken time-reversal symmetry [[46], [48], [49]]. Unfortunately, it is extremely challenging to find available magneto-optical materials at visible and near-infrared frequencies for integrated photonic devices [50]. In contrast, VPCs provide a practical approach to address the backscattering issue induced by certain defects. However, multiple Brillouin zone folding strategy in VPCs cannot guarantee the continuous broadband slow light due to the mismatched bulk symmetry at the topological boundary, resulting in the occurrence of multiple mini-gaps [[51], [52]] and the discontinuous mode dispersion when two non-orthogonal valley kink modes cross. So far, there have been no reports on broadband topological near-flat-band slow light in two-dimensional VPCs.

In this paper, we proposed a scheme to generate broadband slow light in topologically protected valley photonic crystals based on the periodically loading gradually size-variant dielectric columns at the domain wall, the quasi-continuous increment of resonance frequencies enables bandwidth expansion of valley kink modes with near-flat-band dispersion through Brillouin zone folding. Addressing the unavoidable mini-gaps, we introduced a complementary VPC waveguide structure in which the allowed frequency ranges aligning to mini gaps occurred in the primitive VPC waveguide. The structural difference between the two VPC waveguides lies in merely the size of edge dielectric columns, enabling the valley kink modes based on continuous broadband slow light by two seamless joint VPCs. We also investigated the directionality of valley kink modes in the joint VPC waveguide structures, which can be selectively controlled by the photonic gap effect and the phase difference of paired dipole sources. Our results reveal promising opportunities towards a plethora of on-chip slow light applications, such as optical switching and buffering, phase shifters [53], and highly sensitive sensors.

## 2 Method

The band structures and eigen modes of VPCs were calculated using finite element method (FEM) method and eigen frequency analysis. The extended supercell consists of five size-disturbed supercells along the x direction and the periodic boundary conditions are applied. The scattering boundary conditions along the y direction are applied. For the calculation of the different types of waveguides propagation, we simulate a numerical domain formed by fifteen supercells in the x (propagation) direction and eight unit cells in the y direction and set the scattering boundary conditions in the x and y direction. We place a circular polarized dipole source at the center of the honeycomb lattice and then get the transmission of different types waveguides.

## 3 Results and discussion

**Fig. 1:**
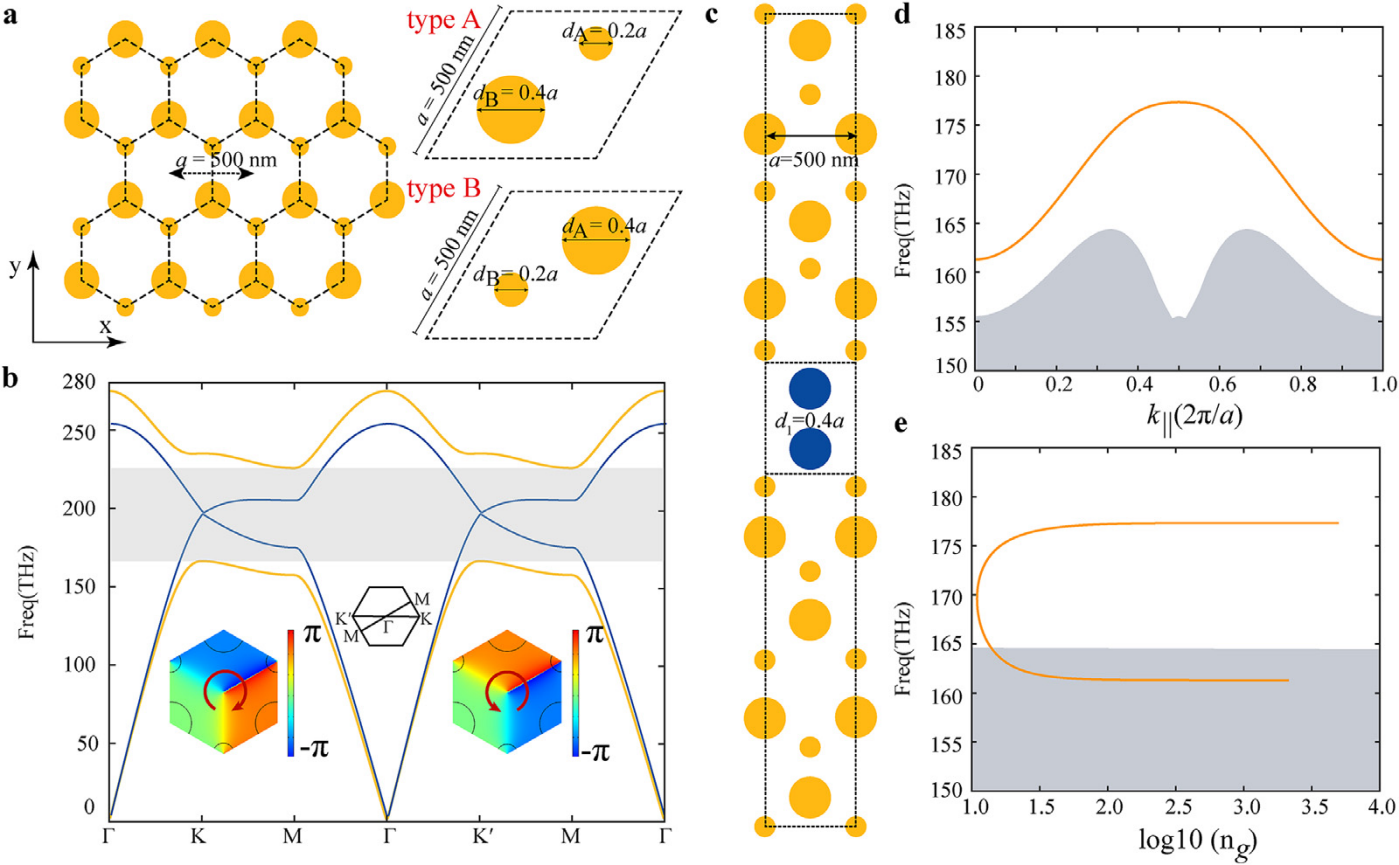
**Geometry and bandstructures of valley photonic crystals.** (a) Schematic of VPCs with a honeycomb lattice (left panel), unit cell of VPCs (type A and type B in right panels). (b) Band structure of VPCs with (blue curves, $d_{A}=d_{B}=0.3a$) and without (yellow curves, $d_{A}=0.4a\;,d_{B}=0.2a$) inversion symmetry. A complete band gap is found between the first and second bulk bands. The insets of (b) are phase distributions of Ez of bulk modes of VPC at the K and K’ point. (c) The schematic of unit cell in VPC waveguide. We outline the cylinder at the topological termination with a dashed line and blue color, with a diameter marked as $d_{1}$, here $d_{1}=0.4a$. (d) The band structure of valley kink mode. The gray area indicates the frequency range where bulk modes coexist. (e) The group refractive index of kink modes varying with versus frequencies. The gray rectangle indicates the frequency range of bulk bands.

Two-dimensional valley photonic crystals is the prototypical structure investigated in this paper, which breaks the spatial inversion-symmetry but preserves the time-reversal symmetry. Transverse-magnetic (TM) polarized photonic band structures are analyzed hereafter which is defined as a nonzero out-of-plane of electric fields ($E_{z}$) [54]. The left panel in Fig. 1a presents the valley photonic crystals where silicon nanopillars (yellow circles) are patterned in a honeycomb lattice embedded in an air background with a lattice constant a. The relative permittivity of silicon is $\varepsilon_{\text{silicon}}=12$ and the background air permittivity is $\varepsilon_{\text{air}}=1$, respectively. Two nanopillars have the same diameter in the unit cell of honeycomb PhCs, $d_{A}=d_{B}$, indicating that the system possesses $C_{6v}$ symmetry and supports a symmetry-protected gapless band structure between the first and second lowest energy bands. The right panel in Fig. 1a represents VPCs with unequal nanopillars in a unit cell, *i.e.*, $d_{A}\neq d_{B}$, indicating the broken inversion symmetry but preserved three-fold rotational symmetry. In Fig. 1b, Dirac point, a hallmark of honeycomb PhCs, is observed at the $K/K^{\prime }$ points in the first Brillouin zone of TM bandstructures (blue curve). For VPCs, the broken inversion symmetry, *i.e.*, $d_{A}\neq d_{B}$, lifts the degeneracy and opens a bandgap, and the frequencies range from 166.3 THz to 225.7 THz (a=500 nm), as the highlighted gray area shown in Fig. 1b. There exist the singularity points of the phase vortex at the K and K’ points with the ±1 topological charges, *i.e.*,the clockwise and counterclockwise phase variation depicted with the argument of the electric field, as shown in the insets of Fig. 1b. Here we define topological charge as $l=\oint_{L}\nabla \left[\text{arg}\left(E_{z}\right)\right]d\vec{s}/2\pi$, where L is a closed contour surrounding the unit cell center. Fig. 1c shows a schematic of super cell in VPC waveguide investigated here. The structure consists of two topologically distinct VPCs, with type A and type B (as shown in Fig. 1a) interfacing with each other. Therefore, the topological invariant is different between the K and $K^{\prime }$ valleys as well as between type A and type B VPCs. Corresponding valley Chern numbers for the first band around K valley are −1/2 and +1/2 for type A and type B VPCs, respectively [54]. By splicing two VPCs with opposite valley Chern numbers, topological kink modes can be observed along the domain walls whether in zigzag edges or beard edges [[42], [55], [56]]. Here we construct a zigzag edge between two types of VPCs. According to bulk-edge correspondence [57], the valley Chern number difference $\Delta C_{v}^{K}=C_{K}-C_{K^{\prime }}=1$ and $C_{v}^{K^{\prime }}=C_{K^{\prime }}-C_{K}=1$ indicates there exists only one kink mode per valley between two bulk domains. Fig. 1c shows the schematic of a super cell of VPCs with δd=−0.2a (here $\delta d=d_{A}-d_{B}$, lattice constant a = 500 nm, $d_{A}=0.2a\;,d_{B}=0.4a$) at the top VPC (type A) and one with δd=0.2a at the bottom VPC (type B). The bandgap size of VPC is proportional to δd. The cylinders at the topological termination distinguish them from the color of the bulk mode region, with a diameter marked as $d_{1}$, where $d_{1}=0.4a$. To find valley kink modes, we calculate the corresponding projected band dispersion where the gray line represents the bulk bands and the orange line shows the dispersion of valley kink modes, as shown in Fig. 1d. Additionally, the frequency range of the complete bandgap spans from 164.4 THz to 223.7 THz. The valley kink mode frequency range extends from 164.4 THz to 177.3 THz, yielding a frequency difference of 12.9 THz. The corresponding valley kink mode wavelength ranges from 1690.88 nm to 1823.56 nm, exhibiting a relative bandwidth of 21.75% for the total bandgap. Topological slow light with a large bandwidth is observed. To illustrate the characteristics of slow light of kink mode, we introduced the group refractive index of valley kink modes, defined as $n_{g}=c/v_{g}$, where c is the speed of light in a vacuum and the group velocity defined as $v_{g}=\partial \omega /\partial k$, with ω is the angular frequency and k is the wavenumber. $n_{g}$ is the key performance indicator for slow light applications and it determines the time and intensity of light-matter interaction. Since the group velocity is the slope of the dispersion, the maximum $n_{g}$ can only be obtained at the point where $k_{∥}=0\;,\pi /a\;,2\pi /a$. Extracted from the bandstructures in Fig. 1d, e shows the frequency dependence of $n_{g}$ and the maximum value reaches 3,000 at the resonance, while in theory, $n_{g}$ at the symmetry points is infinite. However, the propagation robustness of slow light at low frequencies (161.3 THz-164.4 THz) is limited due to its overlap with the bulk mode frequency.

**Fig. 2:**
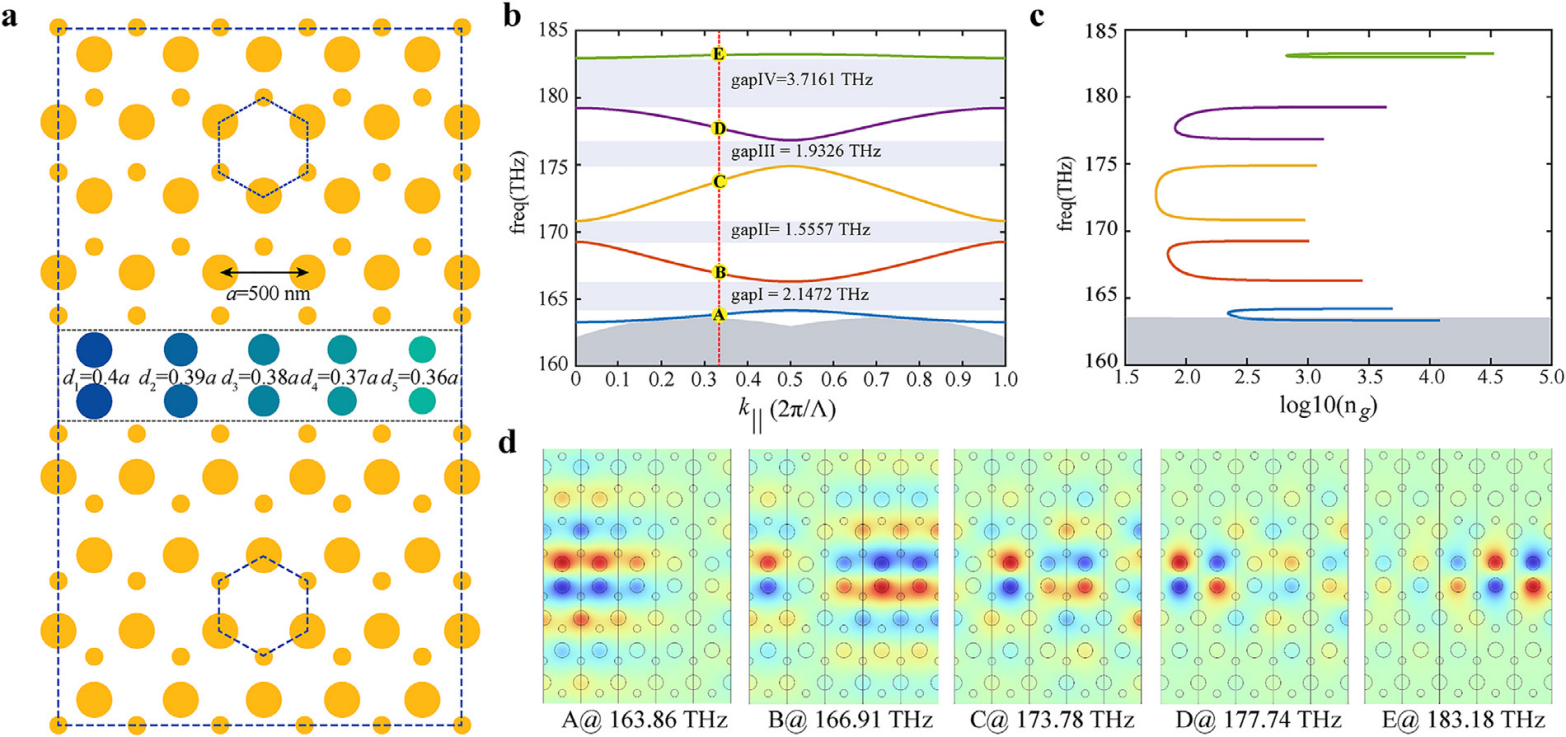
**Bandstructure and eigenmodes property of VPCs with size-perturbed Si nanopillars near the zigzag edge.** (a) The schematic of the VPCs. The diameters of the gradient-colored nanopillars at the zigzag positions are 0.4a, 0.39a, 0.38a, 0.37a, and 0.36a, respectively. (b) Projected band structure of the proposed VPC. The dark gray area indicates the frequency range where bulk modes coexist and light gray areas highlight the mini gaps. (c) Group refractive index of the valley kink modes corresponding to bands shown in (b). The gray rectangle indicates the frequency range where bulk modes coexist. (d) Electric field distribution with changing frequencies, points from A to E are selected at fixed $k_{∥}=\left(2\pi /3\right)/\Lambda$ in (b).

We focus on achieving broadband slow light by folding the valley kink mode dispersion around the Brillouin zone multiple times, in order to maintain a large average group index over a broad frequency range. For the purpose of achieving broadband slow light, we introduce size-perturbation of the nanopillars at the topological domain wall to engineer mode dispersion. Therefore, by varying the diameter and increasing the number of nanopillars near the zigzag edge, we develop the extended supercell composed of five supercells, which has the gradually changed diameters with the step of 0.01a at the domain wall, represented with gradient colors in Fig. 2a. In this context, we refer to the waveguide with extended supercell containing five supercells as primitive waveguide (see the results of different numbers of size-perturbed-nanopillars and physical insight of broadband topological slow light in the Supporting materials). In Fig. 2b, the projected band structure of the VPCs is discontinuous at $k_{∥}=0\;,\pi /\Lambda \;,2\pi /\Lambda$, (here Λ=5a), when two valley kink modes encounter, resulting in the occurrence of four mini gaps [[51], [52]]. The valley kink mode frequency range extends from 164.4 THz to 183.2 THz, yielding a frequency difference of 18.8 THz, exhibiting a relative bandwidth of 31.7% for the total bandgap. The plot of group index varying with frequencies is illustrated in Fig. 2c. Despite the discontinuous bandstructure limits some slow-light applications, the introduction of size-gradient nanopillars enables the further reduction of group velocity and enlarged delay-bandwidth product for valley kink modes. To understand the connection between the size of nanopillars and near-flat band frequencies, we examine the distribution and variation of electric fields during the evolution of kink modes. In Fig. 2d, when $k_{∥}=2\pi /3\Lambda$, the corresponding eigenmode distribution at each band displays the variation of electric field intensity distribution with changing frequencies. For a fixed wavenumber $k_{∥}$ in bandstructure, we observe that the enhanced electric field occurs at the locations with the large (small) diameter of the nanopillars for low (high) frequencies. Additionally, we find that the bonding and anti-bonding modes distribution, an analogue of molecular orbitals also can be used to explain the varying resonant frequencies [58]. (More details about the symmetric and antisymmetric modes are provided in the Supporting materials.) For example, in the lowest and highest bands shown in Fig. 2b, the symmetric field distribution is mainly enhanced at the nanopillars with larger diameters (A panel in Fig. 2d), in contrast, the anti-symmetric field distribution is observed at nanopillars with smaller diameters (E panel in Fig. 2d).

**Fig. 3:**
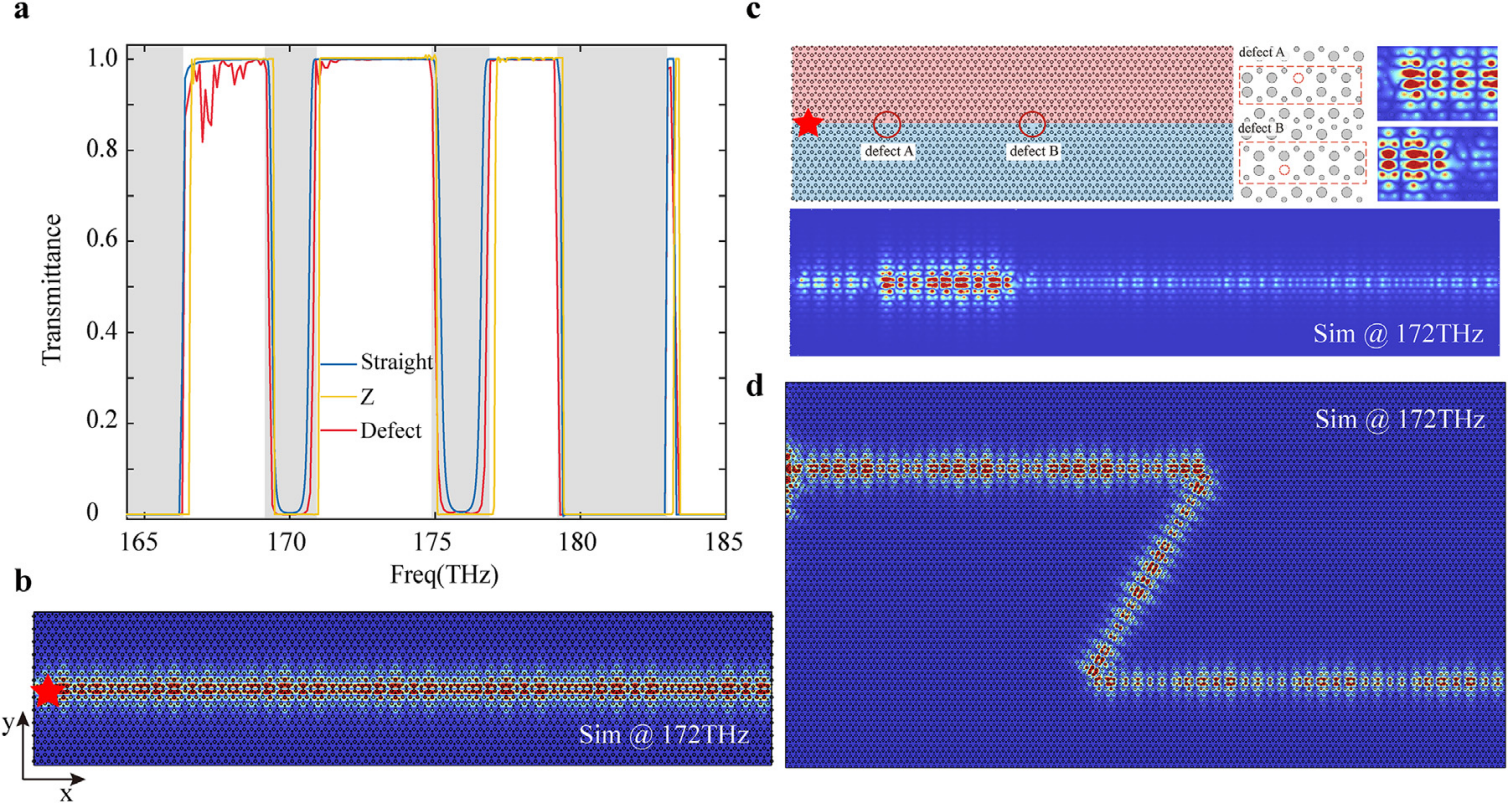
**Verification of topological robustness of kink modes in different types of waveguides.** (a) Transmission spectra of kink modes of straight waveguide (blue curve) and defective straight waveguide of removing two nanopillars at the topological termination (light red curve) and Z-shape waveguide (yellow curve). The gray regions correspond to the mini gap of VPCs. The frequencies range from 164.4 THz to 185 THz covering the entire bandwidth of valley kink modes. (b) Simulated $|E_{z}|$ at 172 THz for the straight waveguide. (c) A schematic diagram of waveguide defects that the red circles mark the removing-nanopillars positions and corresponding electric field distribution pattern at 172 THz. The inset (right panel) shows the electric field distribution at the defect location. The inset (middle panel) shows the local magnification view of the defects. The red dashed rectangle represents a period of nanopillars, with the red dashed circles indicating the missing nanopillars. (d) Simulated $|E_{z}|$ at 172 THz for the Z-shape waveguide. The Z-shape interface with two $120^{\circ }$ bends shows that light can smoothly propagate around the corners. The position of the electric dipole excitation source is marked by the red pentagram.

Next, to investigate the propagation performance of the valley kink mode, we analyze straight, defected and sharp-bended VPCs waveguides. An electric point dipole linearly polarized along the x direction near the topological termination is used as the excitation source. The corresponding transmission spectra for three types of one-way waveguides are illustrated in Fig. 3a, with the frequencies ranging from 164.4 THz to 185 THz covering the entire bandwidth of valley kink modes. The transmission is defined as $T=P_{out}/P_{in}$, the Poynting vector integration $P_{in}$ and $P_{out}$ represent the amount of light transmission that is coupled to the input and output port propagating valley-dependent edge states, respectively. Within the mini-gap region (indicated by the gray bar), the transmission is forbidden with zero transmittance, consistent with the results of the bandstructure. Within the kink mode frequencies region, the transmission spectra remain stable on the flat-top high-transmittance platform, even when subjected to a Z-shaped interface. Fig. 3b depicts the electric field intensity of a straight waveguide at 172 THz, which is composed of 20 extended supercells and has a length of 50 μm along the x direction. To assess the topological robustness against structural disorders near the edge termination, we design a type of point defect with the removal of two nanopillars near the zigzag edge positions, as red circles shown in Fig. 3c. From the overall electric field distribution and the magnified local electric field viewed in Fig. 3c, field distribution of valley kink modes demonstrates the robust transport against missing nanopillars despite exhibiting a slight resonance enhancement at the missing position. The total transmission is not affected by the defects allowing it to maintain a high transmission performance. We then construct a “Z” shaped domain wall to demonstrate robust valley edge transport in the absence of inter-valley scattering. The simulated electric field pattern confirms that the propagating light at 172 THz will smoothly detour by $120^{\circ }$ bending, as shown in Fig. 3d. The transmission of “Z” shape waveguide also maintains a high transmission performance. We can see the same transmittance for the Z-shaped bend as the flat channel within the band gap, proving the broadband robustness of topological valley transport.

**Fig. 4:**
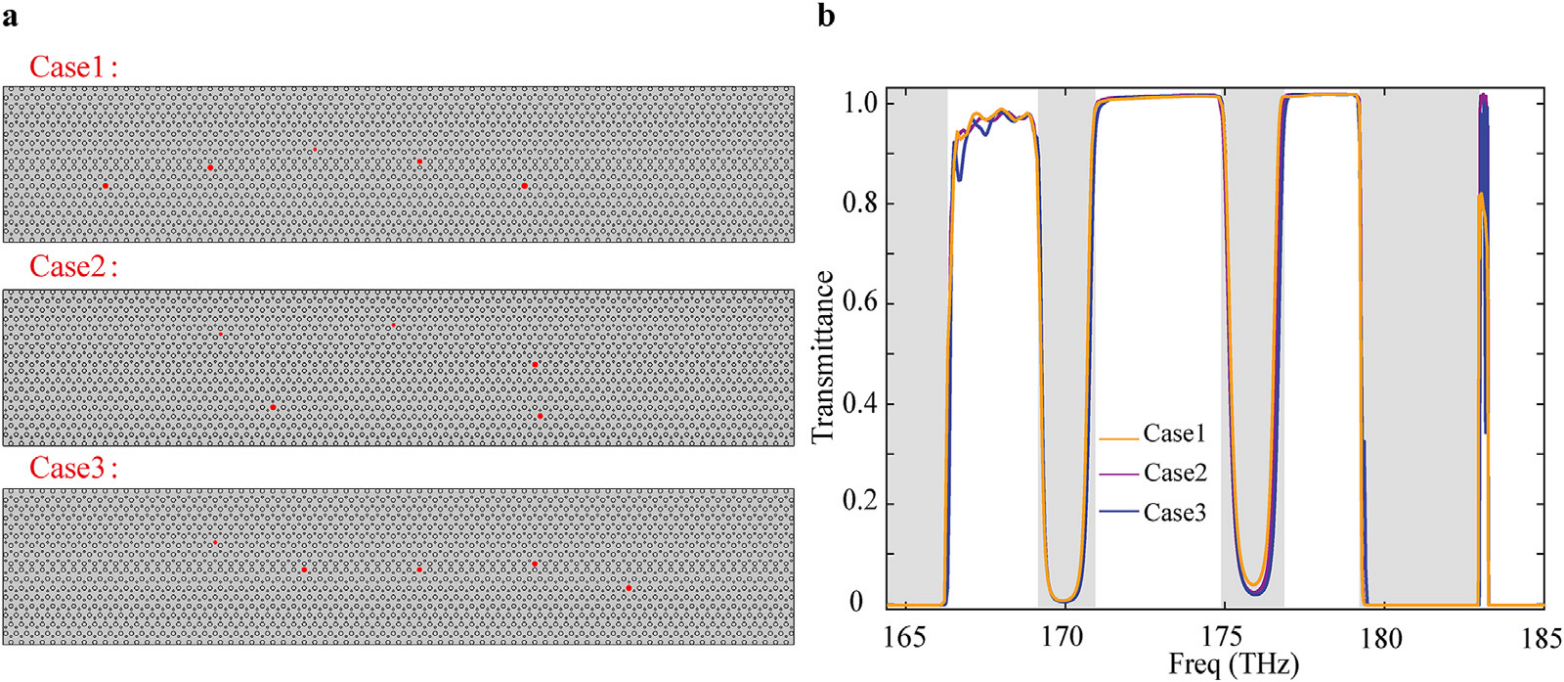
**Schematic diagrams of three disordered waveguides and the corresponding transmittance spectra**. The left panel (a) illustrates the waveguide geometries, with red dots indicating silicon pillars with intentional positional disorder Δr=±20 nm. The right panel (b) displays the transmittance spectra for the three configurations.

To simulate the effect of fabrication imperfection, we randomize the position of the five pillars around their ideal value according to a normal distribution. We introduce disorder in straight waveguides by displacing the position of five pillars independently a random distance from their position in the perfect lattice Δr. This distance is normally distributed with a standard deviation $\sigma =\sqrt{\langle \Delta r^{2}\rangle }$ and 〈Δr〉=0, where brackets indicate the ensemble average over three configurations of random fluctuations. To analyze the position-perturbed waveguide structure, we construct a numerical simulation domain comprising fifteen supercells along the propagation direction (x-axis) and eight supercells along the transverse direction (y-axis), as shown in Fig. 4. Scattering boundary conditions are implemented at all domain boundaries to minimize spurious reflections. The transmittance spectra under these three conditions demonstrate consistently low transmittance within the bandgap regions (T<0.1), while maintaining high transmittance at band frequencies (T>0.9). Therefore, even with a displacement distance Δr=±20 nm, corresponds to 14% of the difference in the center-to-center distances of two adjacent nanopillars, the broadband topological slow-light waveguide still maintains robustness against position disorder defects.

**Fig. 5:**
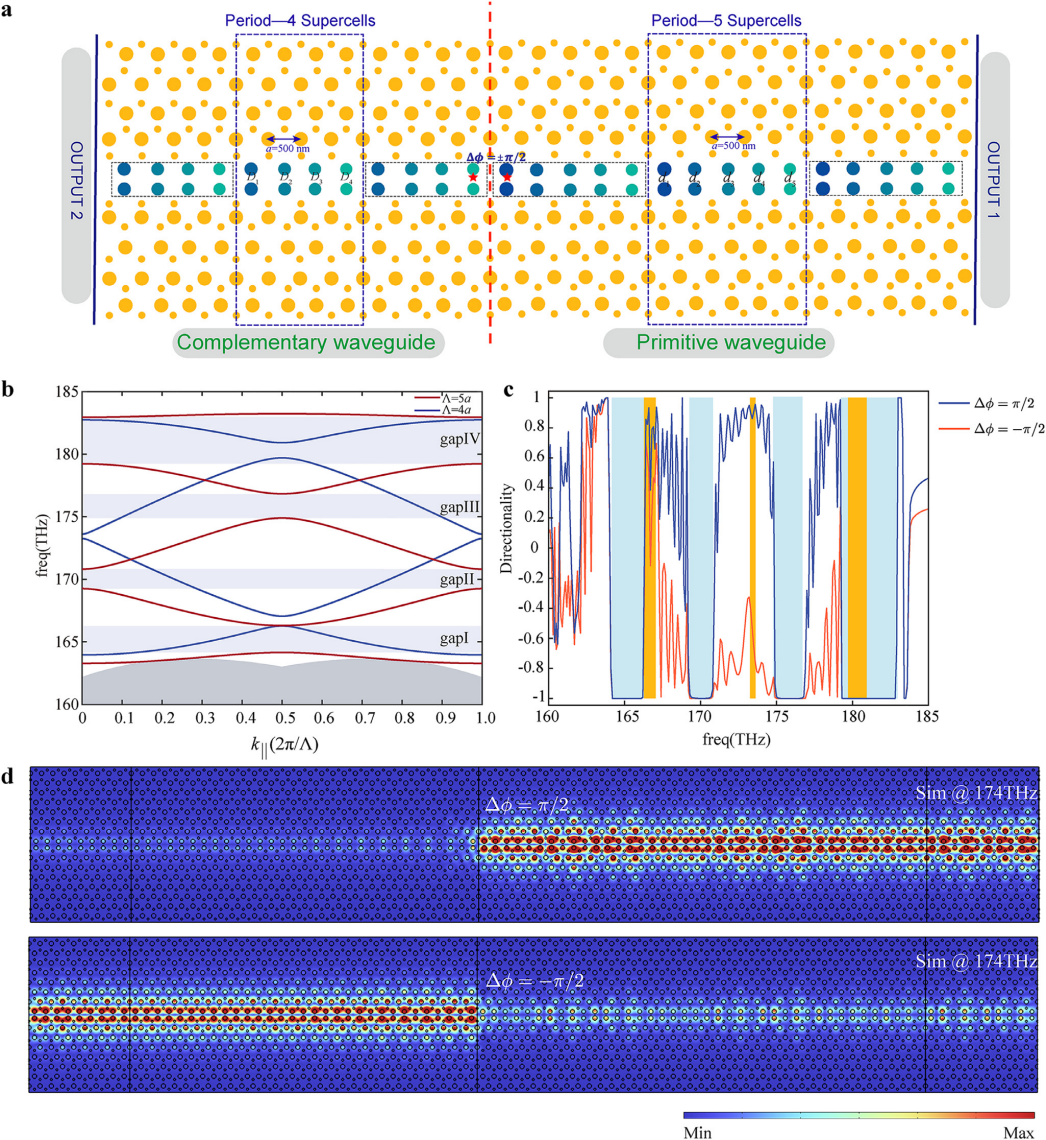
**Frequency-selective topological beam splitter.** (a) The schematic diagram illustrates the overall structure of a frequency-selective topological beam splitter, the primitive waveguide comprising five supercells on the right side and the complementary waveguide comprising four supercells on the left side. And the paired dipole source is positioned at the central location, marked by a red pentagram. (b) The band diagrams of the main structure (indicated by red lines) and the complementary structure (indicated by blue lines). (c) The directionality $D_{x}$ under different phase differences of the point source excitations. The blue region represents the bandgap of the primitive waveguide structure, and the yellow region indicates the bandgap of the complementary waveguide structure. (d) The electric field distribution with flipping dipole source phase difference at 174 THz.

To address the mini gap issue, we introduce a complementary waveguide structure shown in Fig. 5a, where the red dashed line divides the entire structure into two parts: the primitive waveguide structure (extended supercell consisting of five supercells) and the complementary waveguide structure (extended supercell consisting of four supercells). The power output at the right port of the structure is monitored and defined as $P_{R}$, while the power at the left port is defined as $P_{L}$. In order to match the four mini gaps in the primitive waveguide structure, we introduce the complementary waveguide structure consisting of four supercells, to ensure that the number of unit cells is consistent with the number of mini bandgaps. By fixing the lattice constant of the VPCs and only adjusting the diameter of nanopillars at the topological termination, the frequency range of the projected bandstructure is matched to the mini gap regions in Fig. 5b. This approach enables the continuous broadband topological slow light waveguide. Here the diameters at the zigzag edge of the four supercells for complementary waveguide are 0.373a, 0.374a, 0.38a, and 0.383a, respectively. Hence, we achieve a frequency-selective topological beam splitter with high directionality, exploiting unidirectional excitation of valley-contrasting kink modes under different phase differences of two electric point dipoles source excitations. By switching the phase difference between two point dipoles at a distance of a to ±π/2, the directional transmission of light to the left or right port can be simulated, as seen in Fig. 5a. Combined with the valley-contrasting kink modes in the VPCs, this approach effectively enables selective light propagation in specific directions. To characterize the directionality of the topological phase excitation source, we calculated the directionality for a given frequency [59], defined as $D_{x}=\left(P_{R}-P_{L}\right)/\left(P_{R}+P_{L}\right)$. The directionality results are shown in Fig. 5c. Within the bandgap range of the primary structure, $D_{x}=-1$, indicating that all light within the bandgap is forbidden in the primitive waveguide and completely reflected to the complementary waveguide. Consequently, the light within the bandgap frequencies of primitive waveguides can be switched to the complementary structure on the left side, indicating the frequency-selective directional propagation of topological slow light. For the overlapping frequency range, we can achieve selective output through the left or right ports by adjusting the phase difference of the excitation source. As depicted in Fig. 5d, a distinct leftward and rightward transmission performance arises when flipping the phase difference (π/2 to −π/2) between the point sources.

## 4 Conclusion

In summary, we have demonstrated broadband topological slow light by folding the valley kink mode dispersion around the Brillouin zone in an all-dielectric platform. With introducing a complementary structure, the issue of mini gap in VPCs was circumvented and valley-contrasting directional slow-light propagation was achieved in a continuous frequency range. In slow light VPC, we demonstrate the topological robust light propagation in different types of waveguides. We also obtain a novel frequency-selective topological beam splitter and our results provide versatile platform for exploring various applications of broadband topological slow light.

## Data Availability

The data that support the findings of this study are available from the corresponding author upon reasonable request.

## Declaration of competing interest

The authors declare that they have no conflicts of interest in this work.

## CRediT authorship contribution statement

**Min Zhang:** Data curation, Formal analysis, Investigation, Methodology, Software, Visualization, Writing – original draft. **Tianji Liu:** Conceptualization, Data curation, Formal analysis, Funding acquisition, Investigation, Methodology, Project administration, Resources, Software, Supervision, Writing – original draft, Writing – review & editing. **Wei Li:** Conceptualization, Formal analysis, Funding acquisition, Investigation, Methodology, Project administration, Resources, Supervision, Writing – review & editing.

## Appendix A Supplementary materials

Supplementary Data S1 Supplementary Raw Research Data. This is open data under the CC BY license http://creativecommons.org/licenses/by/4.0/ Supplementary Data S1
