# Supplementary material for: Multiple Brillouin zone folding based broadband topological slow light in valley photonic crystals
Source: Fundam Res. 2025 Oct 1;6(2):733–9. doi: 10.1016/j.fmre.2025.09.019 (PMC13069853; doi:10.1016/j.fmre.2025.09.019)
Supplement: Supplementary Data S1 — Supplementary Raw Research Data. This is open data under the CC BY license http://creativecommons.org/licenses/by/4.0/ [file mmc1.pdf]

Supplementary Material

Multiple Brillouin Zone Folding based Broadband Topological Slow Light in Valley Photonic Crystals

Min Zhang[1,2], **Tianji Liu**[1,2,*], **Wei Li**[1,2,*]

[1] GPL Photonics Laboratory, State Key Laboratory of Luminescence Science and Technology, Changchun Institute of Optics, Fine Mechanics and Physics, Chinese Academy of Sciences, Changchun, Jilin 130033, China

[2] University of Chinese Academy of Sciences, Beijing 100039, China

*liutianji@ciomp.ac.cn

*weili1@ciomp.ac.cn

1. The related results of different numbers of size-perturbed supercells

We calculated the band structures and group refractive indices of extended supercells with different diameters at the domain wall. The diameters are 0.4*a*, 0.39*a* (as shown in **Fig. S1a**), 0.4*a*, 0.39*a*, 0.38*a* (**Fig. S1b**) and 0.4*a*, 0.39*a*, 0.38*a*, 0.37*a* (**Fig. S1c**), respectively. As the number of calculated supercells increases, a broader bandwidth and higher group refractive index can be achieved. The trend of bandwidth and average group indices changing with the number of size-perturbed Si nanopillars at the domain wall is shown in **Fig. S1d**. The results are optimal with five size-perturbed supercells and the diameter step of 0.01*a*.

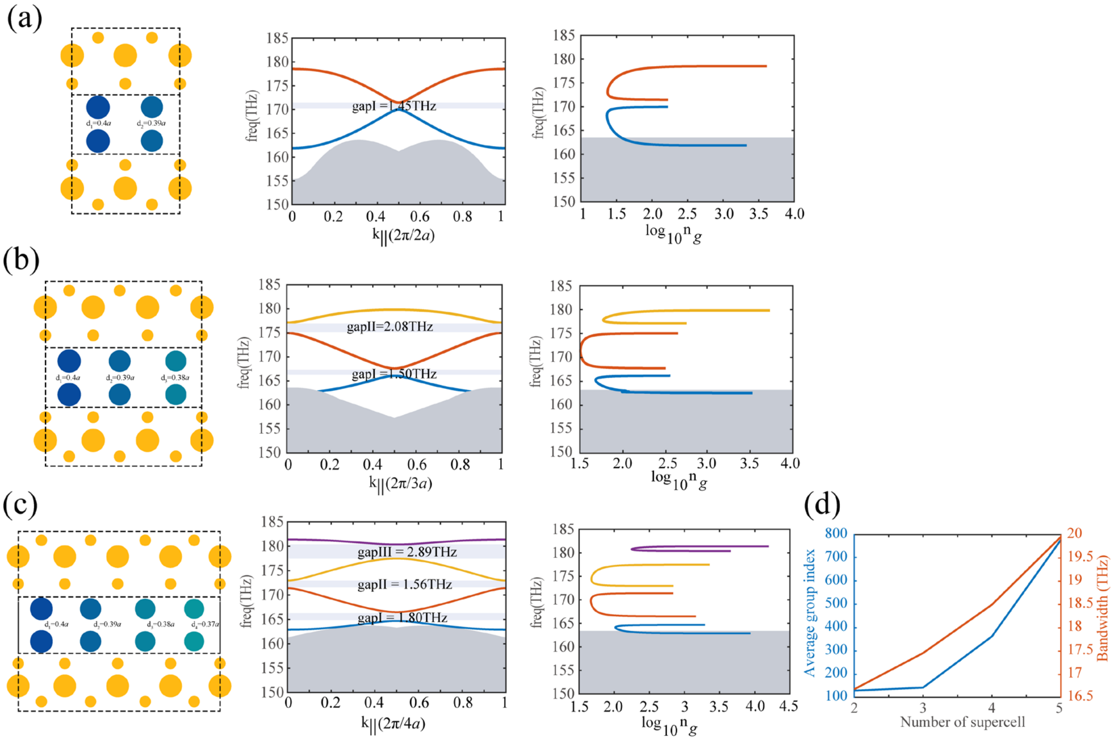

Figure S1. Bandstructure and group refractive indices of VPCs with the different number of size-perturbed Si nanopillars at the domain wall. The left panel illustrates the geometry of extended supercells. The middle panel presents the projected band structure of the proposed VPCs and the right panel depicts the group index of the valley kink modes corresponding to bands. The dark gray area represents the bulk mode, and light gray areas highlight the mini gaps. (a-c) Two, three and four extended supercells with size-perturbed Si nanopillars at the domain wall. (d) The average group refractive index and bandwidth versus the number of supercell.

2. The physics insight of generation of slow-light

The frequency spacing of resonators also have a strong impact on slow-light performance. The enhanced coupling strength reduces the frequency spacing of resonators ($\Delta\omega$), which consequently narrows the bandgap and facilitates the realization of continuous broadband topological slow light. Also, the k-dependent gradient of the coupling strength ($d\Gamma/dk$) determines the modulation rates of n_g and GVD through band structure engineering. Here we calculated the coupling strength between the neighboring bands. At high-symmetry points in the Brillouin zone, the bandgap opening magnitude exhibits a positive correlation with the coupling strength. Therefore, by controlling both the magnitude of coupling strength and its slope variation with respect to wave vector k, we achieve simultaneous tuning of the n_g and GVD, enabling higher n_g and lower GVD.

The coupling between two neighboring bands, is typically analyzed through the mode matching, quantified by the overlap integral of the eigen modes, particularly at mini gap opening regions. The magnitude of the overlap integral between neighboring bands determines the mini gap formation at the high-symmetry points (k=0 and k=π/(5a)) in the Brillouin zone,

$$\Gamma = \frac{\left|\iint E_m(x,y)\cdot E_n^*(x,y)dxdy\right|}{\sqrt{\iint |E_m(x,y)|^2 dxdy \cdot \iint |E_n(x,y)|^2 dxdy}},$$

where E_m and E_n (with band index m and n denoting the two neighboring bands) represents the z-component of the electric field under TM polarization. In Figure S1, we plot the band frequency difference and coupling strength versus the wave vector k. Here the variation of the coupling strength Γ exhibits a precise correspondence with the frequency difference $\Delta\omega(k)$ especially at high-symmetry points. Specifically, for the minigap3 in Figure 2(b) of the manuscript, both $\Delta\omega(k)$ and Γ demonstrate a monotonic decrease as the wave vector k increases. In the system with the time-reversal symmetry, the coupling strength distribution keeps mirror symmetry in the Brillouin zone.

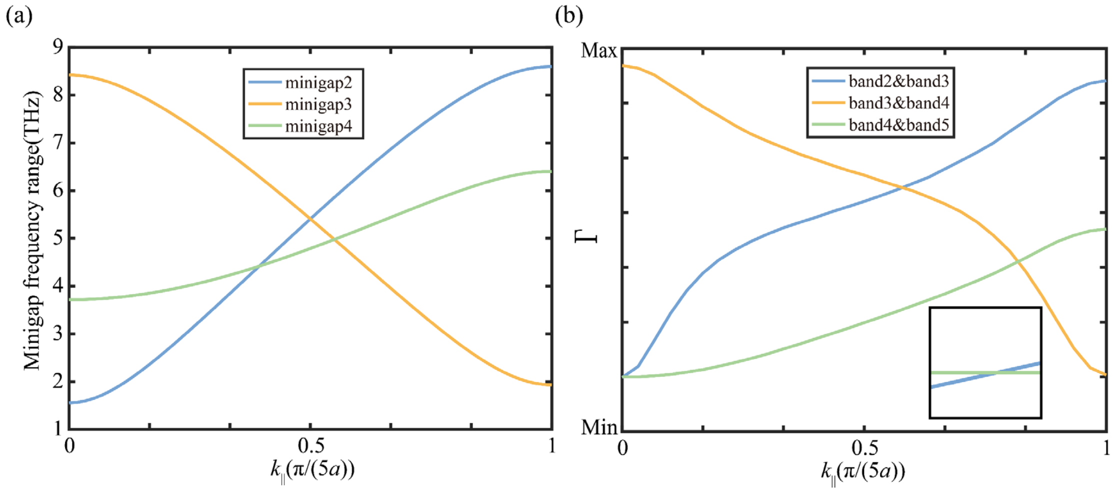

Figure S2. Schematic illustration of the band frequency difference and coupling strength versus the wave vector *k*. (a) The blue, orange, and green curves represent the frequency differences of minigap1, minigap2, and minigap3, respectively. (b) The corresponding curves depict the coupling strength between neighboring bands, specifically band2&band3 (blue), band3&band4 (orange), and band4&band5 (green). The inset provides a magnified view of the blue and green curves at the $k_{\|}=0$.

3. The electric field distribution of symmetric and antisymmetric modes

We calculated the electric field distribution at the symmetry points ($k_{\|} = 0$ or $\pi/(5a)$). At higher frequencies, anti-bonding modes emerge, which are predominantly localized near the smaller-diameter silicon pillars, as indicated by the white arrows in Figure R4. Conversely, at lower frequencies, bonding modes appear, also corresponding to regions with larger diameters of silicon pillars. We have added mode field distributions at the symmetry points ($k_{\|} = 0$ or $\pi/(5a)$), which exhibit similar behavior to those observed at the valley points ($k_{\|} = 2\pi/3/(5a)$). In summary, bonding modes correspond to low frequencies, while anti-bonding modes correspond to high frequencies. Additionally, bonding modes (anti-bonding modes) correlate with the larger (smaller) sizes of the nanopillars. These two factors together result in the correspondence between frequency and eigen mode.

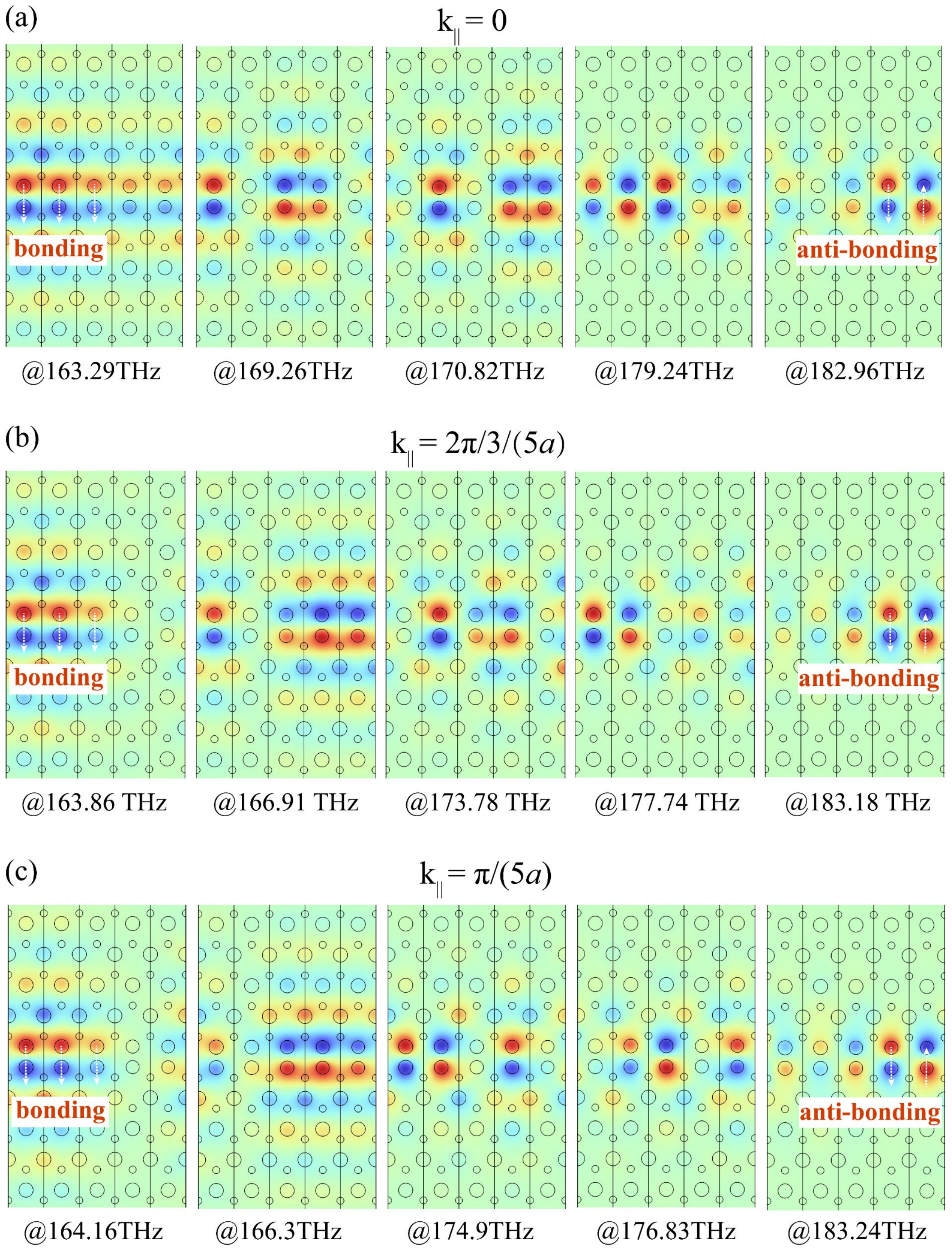

Figure S3. The electric field distribution with changing frequencies from low frequency to high frequency at fixed (a) $k_{\parallel} = 0$, (b) $k_{\parallel} = 2\pi/3/\Lambda$, (c) $k_{\parallel} = \pi/\Lambda$ ($\Lambda=5a$).
